# A Survey of FDG- and Amyloid-PET Imaging in Dementia and GRADE Analysis

**DOI:** 10.1155/2014/785039

**Published:** 2014-03-19

**Authors:** Perani Daniela, Schillaci Orazio, Padovani Alessandro, Nobili Flavio Mariano, Iaccarino Leonardo, Della Rosa Pasquale Anthony, Frisoni Giovanni, Caltagirone Carlo

**Affiliations:** ^1^Nuclear Medicine Department, Vita-Salute San Raffaele University, San Raffaele Hospital and Division of Neuroscience, San Raffaele Scientific Institute, Via Olgettina 60, 20132 Milan, Italy; ^2^Nuclear Medicine Department, University of Rome “Tor Vergata” and IRCCS Neuromed, 86077 Pozzilli, Italy; ^3^Department of Medical and Experimental Sciences, Unit of Neurology, Brescia University, 25123 Brescia, Italy; ^4^Department of Neuroscience Ophthalmology and Genetics, University of Genoa, 16132 Genoa, Italy; ^5^IBFM-CNR, Via F.lli Cervi 93, Segrate, 20090 Milan, Italy; ^6^IRCCS Centro San Giovanni di Dio Fatebenefratelli, and Memory Clinic and LANVIE, Laboratory of Neuroimaging of Aging, University Hospitals and University of Geneva, 1225 Geneva, Switzerland; ^7^University of Rome Tor Vergata and IRCSS S. Lucia, 00142 Rome, Italy

## Abstract

PET based tools can improve the early diagnosis of Alzheimer's disease (AD) and differential diagnosis of dementia. The importance of identifying individuals at risk of developing dementia among people with subjective cognitive complaints or mild cognitive impairment has clinical, social, and therapeutic implications. Within the two major classes of AD biomarkers currently identified, that is, markers of pathology and neurodegeneration, amyloid- and FDG-PET imaging represent decisive tools for their measurement. As a consequence, the PET tools have been recognized to be of crucial value in the recent guidelines for the early diagnosis of AD and other dementia conditions. The references based recommendations, however, include large PET imaging literature based on visual methods that greatly reduces sensitivity and specificity and lacks a clear cut-off between normal and pathological findings. PET imaging can be assessed using parametric or voxel-wise analyses by comparing the subject's scan with a normative data set, significantly increasing the diagnostic accuracy. This paper is a survey of the relevant literature on FDG and amyloid-PET imaging aimed at providing the value of quantification for the early and differential diagnosis of AD. This allowed a meta-analysis and GRADE analysis revealing high values for PET imaging that might be useful in considering recommendations.

## 1. Introduction

In Western countries, during the last century, the elderly population (over 65) has almost triplicated and in the next fifty years it will represent almost 35% of the total population. Along with ageing, dementia will become not only a dramatic clinical entity, but also a serious socio-economic issue, given that patients diagnosed with this devastating disease will likely increase by 50% by 2030.

However, the 2011 World Alzheimer Report (http://www.alz.co.uk/research/world-report) has underlined that only a percentage ranging between 20 and 50% of dementia cases are identified and recognized in the early stages, that is, at least half of the population of dementia patients suffering do not receive a complete diagnostic workup since disease onset.

This diagnostic delay gives rise to a so-called “treatment gap” between early stages of the disease and a formal diagnosis which can then trigger necessary care and organized support ameliorating the patient's quality of life along with that of caregivers and family members. Clinical diagnosis* per se* has limited accuracy and requires the presence of cognitive symptoms, while biomarkers that are specific for AD-related pathologic phenomena would allow more accurate diagnosis when patients are in the prodromal or even preclinical stage of the disease, a period that is generally held to be the best intervention time for AD, at least at present days. PET allows the investigation of both the measurements of cerebral glucose metabolism by ^18^F-2-fluoro-2-deoxy-D-glucose (FDG) and the A*β* amyloid deposition through specific molecular imaging techniques involving radiopharmaceuticals binding to amyloid. In the last decades, PET evidence for functional and molecular changes in neurodegenerative diseases has been largely shown [1–4]. In Alzheimer's disease (AD), within the two major classes of biomarkers now identified, biomarkers of disease state (i.e., biomarkers of amyloid *β* [A*β*] accumulation) and biomarkers of disease stage (i.e., biomarkers of neuronal injury), amyloid-PET, and FDG-PET imaging represent critical and decisive tools. PET imaging is now recognized of value to the early diagnosis and to clearly support the final diagnosis of AD [5–8]. Revisions of the NINCDS-ADRDA diagnostic criteria of AD [5, 9], as well as the new National Institute of Aging-Alzheimer Association criteria of MCI due to AD [6] have been proposed, positing that individuals with memory impairment who are positive for AD biomarkers have a high likelihood of having AD pathology. The corollary is that biomarker positive MCI patients frequently progress to dementia. Crucially, when both Abeta and neuronal injury biomarkers are negative, the dementia is unlikely to be attributable to AD pathology [1, 10–12].

The references based recommendations rely on sensitivity and specificity of the PET methods derived by the imaging literature that is based either on parametric approaches or on visual method that greatly depends on the observer's experience and lacks a clear cut-off between normal and pathological findings.

On the other hand, PET neuroimaging research has focused on the development of tools improving either detection of people at higher risk of dementia or early diagnosis of Alzheimer disease (AD) [13–16]. These methods improve the accuracy for the diagnosis of AD and prediction of progression from mild cognitive impairment to AD dementia [17–23]. Noteworthy, markers of amyloidosis and neurodegeneration are currently being used as outcomes in proof-of-concept drug studies [24].

The sensitivity and specificity of the PET methods indeed greatly depends on the use of quantification methods [15, 25, 26]. For example, FDG-PET can be assessed using software that analyses the pattern of tracer uptake voxel-wise by comparing the subject's scan with a reference data set of normal ageing, allowing a better recognition of the patterns of hypometabolism compared with visual interpretation [15, 17, 27].

The same is true for measurements of amyloid load using PET [25, 28, 29]. In AD, it has been shown that quantification or parametric measurements of amyloid load are fundamental since they allow cut off scores for a better differentiation between normal subjects, preclinical AD, and AD individuals [21, 22, 30]. In addition, due to the demonstration between group and intersubject variability, quantification of amyloid load would be crucial for multicentre studies and therapy monitoring. A real problem exists, whether a dichotomous readout such as that of amyloid-PET scans will be used (or misused) in the diagnostic procedures. It needs to be prevented a positive amyloid scan to become a* de facto* diagnosis of AD. Semiautomated (such as standardized uptake value ratio (SUVR)) or automated semiquantitative measures (such as using SPM-based protocols) will have the advantage of being operator independent. Semiquantitative or quantitative measures require thresholds for positivity/negativity. Thresholds include information on risk to develop dementia for subthreshold degrees of amyloid positivity. Semiquantitative or quantitative measures might in the future discriminate “accumulators” from “nonaccumulators,” distinction that in normal persons could predict the development of MCI as a prodromal step to full blown AD [31]. Finally, it has to be highlighted that, today, the rationale for the use of PET biomarkers in prodromal AD diagnosis is that biomarkers change over decades before full-blown AD dementia develops [32].

Aim of this paper was to provide a survey of the specific PET literature based on the above considerations, with a meta-analysis and a GRADE analysis on FDG- and amyloid-PET imaging in the early and differential diagnosis of Alzheimer disease.

This survey was based indeed on restricted inclusion criteria of the relevant literature, namely,

1. only articles published since 2001 which retain high quality 3D PET scans and control to an optimal degree any methodological shortcoming;
2. for FDG-PET, only studies employing voxel-based analysis techniques (such as SPM, Neurostat, and AD t-sum) with statistical parametric mapping procedures that can provide unbiased, statistically defined measures of brain abnormality in the individual brain toward a reference control population throughout the whole brain;
3. specifically to amyloid-PET, only articles reporting quantification or parameterization of *β*-amyloid deposition (in AD, MCI subjects, and normal controls) either with short half-life ^11^C-labeled ligands (^11^C PIB) and ^18^F-labeled tracers (^18^F-AV-45 Florbetapir, ^18^F-BAY94-9172 Florbetaben, and the ^11^C-PiB derivative ^18^F-GE-067 Flutemetamol).

In addition, we included a descriptive analysis of the related literature reporting differences in the levels of sensitivity and specificity for the standard visual FDG-PET scan or dichotomous readout based amyloid-PET with respect to parametric or semiquantitative analysis [33–35].

### 1.1. Premises on FDG-PET Imaging Studies

^18^F-Fluorodeoxyglucose-PET (^18^F-FDG) is used to measure cerebral metabolic rate of glucose that is considered an index of synaptic functionality and density [36]. It has been widely used for various purposes, ranging from early diagnosis to differential diagnosis of dementias [3, 4]. There is substantial agreement about its effectiveness for diagnosis of dementia mainly for the typical hypometabolism patterns associated with the different neurodegenerative conditions (see [16]). Hypometabolism in AD has showed a very peculiar pattern since the emergence of early PET evidences [37, 38] recently defined in detail as involving parietal and temporal regions, precuneus, posterior cingulate cortex, medial temporal cortex, and structures (like hippocampus) [10, 14, 39–41]. Cerebral map of glucose metabolism can be visually inspected by experienced raters to evaluate possible neurodegenerative patterns. Despite the potential of visual inspection, modern techniques for quantification of FDG uptake are now widely used, and have been demonstrated to improve diagnosis accuracy and readability of hypometabolism patterns [33]. Statistical parametric mapping (SPM) produces unbiased smoothed and regularized images that allow a comparison between a single patient and a control group to define functionally abnormal regions. ^18^F-FDG has been otherwise widely used to differentiate AD from non-AD dementias like DLB or FTLD spectrum. In a landmark study, Minoshima and coworkers [42] reported that relying on occipital cortex metabolism produced a sensitivity of 90% and a specificity of 80% in discriminating AD versus DLB, using autopsy pathology as reference. Similarly, Foster et al. [33] showed that ^18^F-FDG can help discriminate between AD versus FTLD spectrum with 97% sensitivity and 86% specificity (93% accuracy). Importantly, studies have been also underlying that an absence of peculiar hypometabolism patterns may exclude a diagnosis of dementia [1].

As a matter of fact, hippocampal hypometabolism, a crucial marker of AD, is often missed, particularly in voxel-based analysis using smoothing procedure. As suggested in literature [41], by using manual region-of-interest-based (ROI) analytical methods and MRI/PET coregistration methods, the temporal medial dysfunction should be highlighted. In addition, even if has to be clarified, the method-related nature of this MRI/PET inconsistency, using coronal and/or sagittal dimensions (anterior-posterior) instead of axial orientation (inferior-superior) may at least partially overcome this “hippocampal issue,” as this formation is smaller in axial view rather than in coronal or sagittal [41].

It appears that the normalization and smoothing procedures of SPM package tool that is necessary to minimize between individual inhomogeneity in brain shape and dimension may mask reduced uptake in small structures, such as the hippocampus. Moreover, spatial resolution of PET systems is best in superficial cortical areas close to the detectors while it is worst in midline and medial structures far from the detectors. Lastly, a pathophysiological explanation admits that the high synaptic density at posterior temporal-parietal association cortex and limbic cortex makes it easier to detect glucose hypometabolism in these regions as compared to the MTL structures which are rich in cell bodies but relatively poorer in synaptic density [43].

Furthermore, another florid field of research regards longitudinal studies to predict MCI-AD conversion and therefore early diagnosis of AD. Different techniques (MRI, PET, CSF, and clinical evaluation) have been extensively compared, and even though combined predictors are now considered the best solution, it has widely reported a major role (namely, in sensitivities, specificities, and prediction accuracy) of the PET [44–47].

### 1.2. Premises on Amyloid-PET Imaging Studies

*β*-amyloid plaques are a hallmark of AD and can be found in moderate to high number in cortical gray matter in all cases of AD and develop many years before the onset of dementia. The amyloid theory postulates that amyloid accumulation is the main causative event leading to synaptic and neuronal degeneration and subsequent gray matter atrophy [31]. This hypothesis is supported by the evidence that the soluble form of *β*-amyloid in equilibrium with the soluble *β*-amyloid found in plaques is potentially neurotoxic though the time interval between the deposition of *β*-amyloid and the beginning of a neurodegenerative process that still remains unclear [48].

In contrast, A*β* plaques are not found in frontotemporal dementia (FTD) or pure vascular dementia [12]. The amyloid hypothesis is still debated and several arguments point against amyloid as a main pathogenic factor in AD pathology [49]. Whatever the role of amyloid is, whether causative or merely an epiphenomenon, all patients with AD have an increased brain amyloid load. Therefore, the development of imaging tools for the detection and quantification of amyloid deposition is of particular relevance for the confirmation or exclusion of AD, the distinction of AD from other dementias, and its early diagnosis [50].

The first tracer for amyloid was developed at the University of Pittsburgh through modification of thioflavin T; a fluorescent dye used to identify plaques in brain tissue specimen [51] that was given the name Pittsburgh compound B (^11^C-PiB). ^11^C-PiB was found to bind to the amyloid in the classic (i.e., neuritic) plaques of AD, which are distributed around the degenerating neuritis. ^11^C-PiB could label *β*-amyloid in living brains, and it was used in patients suffering from AD since the earliest investigations [52]. It lacks specificity to these classic plaques, as it also binds to diffuse amyloid plaques that can be found in a substantial proportion of healthy elderly and are not specific for AD [53]. Further, PiB binds to cerebrovascular amyloid in cerebral amyloid angiopathy (CAA), mainly in posterior parietal and occipital cortex. As such, PiB cannot be regarded as a specific marker of AD-amyloidosis but rather of brain amyloidosis more in general.

Leinonen et al. [54] evaluated ^11^C-PiB uptake findings in AD patients with and without typical AD neuropathological lesions in frontal cortical biopsy specimens. The authors found a significantly higher PiB uptake in the frontal, parietal, and lateral temporal cortices and striatum in patients with A*β* aggregates in the frontal cortex compared with those without notable A*β* aggregates in the brain biopsy specimen. Moreover, the patients with the highest A*β* load in the biopsy specimen had also the highest ^11^C-PiB uptake in PET imaging.

Several authors investigated the diagnostic accuracy of AD by means of ^11^C-PiB PET as unique imaging method or in combination with other measures (usually FDG-PET or volumetric MRI) and mainly using clinical criteria as reference test. For example, by comparing ^18^F-FDG to ^11^C-PiB PET scan, Lowe et al. [55] obtained a similar diagnostic accuracy in early cognitive impairment, but ^11^C-PiB PET scan allowed a better discrimination between amnestic MCI and nonamnestic MCI, thus demonstrating that amyloid deposition occurs before cerebral metabolic dysfunction.

Devanand et al. [56] found that ^11^C-PiB binding potential (BP) analysis slightly outperformed regional cerebral metabolic rate for cerebral glucose analysis of FDGPET images in discriminating AD patients from healthy controls (HC).

Similarly, [34] demonstrated the higher sensitivity of ^11^C-PiB BP analysis in discriminating AD from FTD patients. Other two studies, comparing ^18^F-FDG-PET and ^11^C-PiB PET, have concluded that they give complementary information for the early diagnosis and followup of patients with dementia [57, 58]. This is a central issue, since dissociation between metabolic reduction and amyloid deposition has been also shown. In particular, in a 3 and 5 years of follow-up study on MCI and AD patients, Kadir and coworkers found that fibrillar amyloid load progressively increased in MCI patients and was followed by more stable level in clinical AD patients, whereas glucose metabolism started to decline early in MCI patients and became more pronounced in advanced clinical stage [59]. Also, the mismatch between the two imaging modalities was shown in a study investigating the effects of phenserine treatment on glucose metabolism and amyloid load in 20 AD patients [60].

A number of longitudinal studies have argued for the role of ^11^C-PiB tracer in predicting conversion from MCI to AD. For example, it has been shown that, compared to nonconverting MCI patients and healthy controls (HC), MCI patients that converted to AD at clinical followup displayed significantly higher ^11^C-PiB retention, at levels comparable to that of AD patients [61]. Okello et al. [21] found that the 50% of MCI patients showing a positive ^11^C-PiB uptake at baseline converted to overt AD at 1-year followup and had greater ^11^C-PiB retention than nonconverter patients. Similarly, in a 2-year follow-up study, Koivunen and colleagues [62], measuring ^11^C-PiB retention in MCI and control subjects, showed that MCI patients who converted to AD had greater ^11^C-PiB retention in several brain areas, including cingulum, frontal and temporal cortices, putamen, and caudate.

Now, it is widely accepted that ^11^C-PiB PET can provide a quantitative representation of fibrillar deposition amyloid-beta deposition in the brain. Therefore, it is of the utmost importance to develop quantitative methods of amyloid-PET data analysis and that such methods can be standardized and applied across centers.

Analyses of PET images for the quantification of A*β* deposition have been done both qualitatively (e.g., visual analysis of tracer uptake) and quantitatively. In this latter case, analysis of tracer retention requires normalization of the uptake values, to allow inter- and intrasubject comparisons. The standard uptake value ratio (SUVR) normalizes the uptake values to the mean uptake value within a region containing nonspecific binding, usually the cerebellar grey matter. Another method, for example, based on distribution volume ratios (DVRs) and their combination with arterial plasma input, metabolite correction, or references tissue models may yield different results [63].

The interrater reliability of manual and automated ROI delineation for ^11^C-PiB PET imaging was recently assessed for the detection of early amyloid deposition in human brain [64]. Despite methodological differences in the manual and automated approaches, the analysis revealed good agreement in primary cortical areas and the cerebellar reference region for SUV and SUVR outcomes. These data are important because a reliable methodology is needed for the detection of low levels of amyloid deposition on a cross-sectional basis and small changes in amyloid deposition on a longitudinal basis and also to enable valid definition of amyloid positivity thresholds and determination of relationships between* in vivo* PET imaging and postmortem assessments of amyloid-beta load.

A new noninvasive efficient graphical approach, called the relative equilibrium-based (RE) graphical plot, has been developed for tracer kinetics analysis, with equilibrium relative to input function; this method has been recently used to improve and simplify two of the most common approaches for ^11^C-PiB PET quantification [65]. In this paper, results from theoretical analysis were confirmed by 78 PET studies of nondemented older adults, indicating that the RE plot could improve pixel wise quantification of amyloid-beta burden when compared with 2 frequently used methods like the Logan plot and the SUVR.

In the majority of ^11^C-PiB PET studies, the cerebellum has been chosen as a reference region. However, because cerebellar amyloid may be present in genetic AD, cerebral amyloid angiopathy and prion diseases, whether the pons could be used as an alternative reference region for the analysis of ^11^C-PIB binding in AD has been evaluated [66]. The findings of the study in 12 sporadic AD patients, 10 age-matched controls, and 3 other subjects (2 with presymptomatic presenilin-1 mutation carriers and one probable familial AD) suggest that that the target-to-pons ratio for the analysis of ^11^C PIB images has low test-retest variability and high reproducibility and can be used as a simplified method of quantification when the cerebellum as a reference is not appropriate.

The definition of a cutoff that separates individuals with no significant amyloid-beta deposition from those in which deposition has begun is crucial for the clinical acceptance of ^11^C-PiB PET. In a cohort of older subjects in which the separation between PiB positive and PiB negative subjects was not so distinct, the application of visual read and quantitative approaches optimized the identification of early amyloid-beta deposition [26].

In addition to ^11^C-PiB, other ^18^F-labeled tracers have been developed and investigated. Flutemetamol (GE-067) is the 3′-fluoro-derivative of PiB, whereas florbetaben (BAY-94-9172, AV-1) and florbetapir (AV-45) are stilbene and styrylpyridine derivatives, which exhibit high affinity binding for fibrillary amyloid. Flutemetamol kinetic analysis of tracer binding showed reliable quantification by use of relative standardized uptake value ratios with the cerebellar cortex as a reference region, and data acquisition for this analysis requires only 20 min scanning and is feasible in a standard clinical setting [67]. Florbetaben and florbetapir are chemically closely related compounds but the former has slower kinetics, resulting in a longer imaging acquisition time (for stable uptake up to 130 min after injection), in comparison with Flutemetamol (90 min) and Florbetapir (60 min) [68].

In a recent PET study using ^18^F-Florbetapir with 74 HC and 29 AD patients with terminal disease, demonstrated a high correlation between* in-vivo* tracer uptake and the presence of *β*-amyloid at autopsy, as well as 96% sensitivity and 100% specificity in distinguishing HC from AD, thus suggesting that ^18^F-Florbetapir PET provides an accurate and reliable assessment of amyloid burden [69]. A large study pooling data from the 4 registered phases I and II trials of florbetapir PET imaging, confirmed the ability of florbetapir uptake analysis to characterize amyloid levels in clinically probable AD, MCI, and HC groups using both continuous and binary quantitative measures of amyloid burden [70].

## 2. Methods

### 2.1. Study Inclusion Criteria

The general inclusion criteria for relevant research studies were the following:

- articles had to be published in a peer-review scientific journal;
- studies reporting sensitivity and specificity measures in relation to a histopathological or clinical diagnosis of neurodegenerative diseases;
- studies including large cohorts of subjects (see Table 1: early diagnosis FDG: range 20–395; Table 2: differential diagnosis FDG: range 45–297; Table 3: early diagnosis amyloid: range 13–107);
- studies investigating the prediction of mild cognitive impairment (MCI) to Alzheimer's disease (AD) conversion that retrospectively analyzed the initial characteristics of those who were progressive and those who remained stable.

#### 2.1.1. Specifically to FDG-PET

(i) Only articles published since 2001 were considered, which retain high quality by controlling to an optimal degree both clinical and methodological shortcomings.

(ii) Only studies employing voxel-based analysis techniques (such as SPM, Neurostat, and AD t-sum) with statistical parametric mapping procedures can provide unbiased, statistically defined measures of brain abnormality throughout the whole brain on a voxel-by-voxel basis; the basic procedure in voxel-based analysis involves the spatial normalization and smoothing of each individual's PET scan to an anatomically defined standard brain reference volume (the template or atlas volume) in the stereotactic space. This enables voxel-by-voxel statistical comparison of the ^18^F-FDG pattern in the individual brain toward a reference control population. FDG uptake in each voxel must be previously normalized to the average uptake of a reference region, since without arterial blood sampling or other validated quantification methods, the standard PET procedure does not allow true quantitative measurements of glucose consumption. The reference region can change; the “default” reference region in SPM is the whole brain while Neurostat allows choosing among the whole brain, the cerebellum, and the thalamus. By changing the reference region, the results of parametric mapping may change as well. Final agreement on the region to be used is still lacking; the choice of whole brain tends to reduce sensitivity because the hypometabolic voxels are included in the average, while the cerebellum tends to increase sensitivity because it is less affected by neurodegeneration in AD. Taking in mind these limitations and that they do not allow true quantitative estimation of glucose metabolism but rather of glucose metabolism distribution, all these procedures result in an observer-independent mapping of regional abnormalities of glucose metabolism.

#### 2.1.2. Specifically to Amyloid-PET

(i) Only articles reporting parameterization of *β*-amyloid deposition in patients with AD, MCI and normal controls either with short half-life ^11^C-labeled ligands ^11^C PIB and ^18^F-labeled tracers (^18^F-AV-45 Florbetapir, ^18^F-BAY94-9172 Florbetaben, and ^18^F-GE-067 Flutemetamol). Articles reporting quantification with other *β*-amyloid compounds have been excluded when (a) there was uncertainty about the selectiveness of the binding to amyloid plaques (e.g., ^11^C BF-227) or (b) utilization of recently released compounds still needing for a systematic evaluation (e.g., ^18^F-AZ4694, namely, NAV4694).

(ii) Furthermore, only articles using quantification methods such as distribution volume ratio (DVR) or standardized volume uptake ratio (SUVR) were included in the analysis. Similar to FDG-PET, to calculate the uptake without blood sampling, results are shown as ratios with a reference region, usually cerebellum (even though utilization of pons is currently debated [66] see also* Pet Amyloid Imaging studies paragraph*). Obviously the change of reference region can affect the results, but as a final agreement is lacking, this is up to the authors to rely on the affinity of the different compounds for multiple reference regions. As regards SUVR, to discriminate between “amyloid positive” and “amyloid negative” burdens (as well as between “low” and “high” retention), authors have been applying cut-off scores, usually obtained by control groups (like in [71] or using values reported in literature i.e., [72] for ^11^C-PIB PET or [73] for ^18^F-Florbetapir). Therefore, manipulating cut-off scores can heavily affect results, leading to radically different groups' characterization. Despite these variations in the methodology of amyloid quantification, automated algorithms can fairly discriminate between different patterns of retention, in an observer-independent fashion, leading to important advantages in clinical practice and diagnosis.

### 2.2. Meta-Analysis and GRADE Analysis

#### 2.2.1. GRADE Evaluation

Scientific evidences available regarding each of the tests (^18^F-FDG-PET or amyloid-PET) for the early and differential diagnosis of AD, as well as for MCI conversion prediction, are graded in terms of Level of Confidence (LoC: VL = very low, L = low, M = moderate, and H = high), as reported by GRADE system [74–76]. Tables 1, 2, and 3 show the level of confidence ratings assigned to the studies reviewed in this paper, indicating that none of the studies was rated high whereas most studies were rated moderate to low.

It is to be mentioned that according to the GRADE system, the best way to assess any “diagnostic strategy” is randomized controlled trials in which investigators randomize patients to experimental or control diagnostic approaches in order to provide high quality evidence of test accuracy for the development of recommendations about diagnostic testing.

Both the clinical context and complex implementation of brain FDG or amyloid-PET protocols, however, paralleled with ethical issues raised by the degree of invasiveness of both procedures, are not comparable to randomized trials or many observational studies in which the alternative diagnostic test has been carried out in order to establish high quality of evidence or clear differences in patient important outcomes based on GRADE framework.

Furthermore, it must be acknowledged that the results of FDG- or amyloid-PET diagnostic approaches do not have nothing to do with effective treatments (as the usual GRADE evaluative study set); however, they may have a significant positive impact in terms of patient outcomes, such as reducing the treatment gap between AD pathological onset and diagnosis of the disease, thus improving ability to plan which can be considered analogous to an effective patient treatment [77]; the correct diagnostic inclusion of patients in pharmacological trials [78], the appropriate family context, and behavior induced by the diagnosis are very useful in supporting pharmacological and cognitive remediation approaches.

Notwithstanding the here selected criteria for investigations employing FDG- or amyloid-PET brain imaging have been rated only as “low” or “moderate” quality evidence for recommendations about diagnostic procedures in a GRADE system, we have to consider that there will be great indirect benefits for their “patient-outcome” (i.e., test accuracy in terms of sensitivity and specificity). Assessing the directness of evidence supporting the use of a diagnostic test requires judgments about the relationship between test results and patient-important consequences, therefore in this paper a severe challenge arose in the attempt to apply GRADE to two crucial questions about FDG- or amyloid-PET as accurate, valid and powerful diagnostic tests, for (1) the early diagnosis and (2) the differential diagnosis of AD.

Guyatt et al. [76] stated that “GRADE will disappoint those who hope for a framework that eliminates disagreements in interpreting evidence and in deciding on the best among alternative courses of action. Although the GRADE system makes judgments about quality of evidence and strength of recommendations in a more systematic and transparent manner, it does not eliminate the need for judgments.”

That is, applying a GRADE system in a PET functional and molecular imaging evaluation for diagnosis can be accepted due to the high value for low and moderate results in such a setting.

In this survey, we performed three different meta-analyses for evaluating the accuracy and effectiveness of diagnostic tests (i.e., FDG or amyloid), in order to make a judgment about quality of evidence (GRADE) on the early or differential diagnosis and for conversion prediction of dementia in our population. Given that the sensitivity of a test shows the proportion of patients with the disease (i.e., AD) whom the test classifies as positive while the specificity shows the proportion without the disease (i.e., no neurodegenerative disease) whom the test classifies as negative, we computed the positive likelihood ratio for each study included in the three meta-analyses, (i.e., FDG-PET or amyloid-PET imaging in the early diagnosis of Alzheimer disease and FDG-PET in the differential diagnosis of Alzheimer disease) which combines information from sensitivity and specificity and gives an indication of how much the odds of disease change based on a positive or a negative result (i.e., accuracy). For example, a positive likelihood ratio of 10 means that a positive test result is ten times more likely in a diseased subject than in a healthy person. The resulting positive likelihood ratio (LR+) for each study was interpreted according to general guidelines for evaluating the probability increase of detecting the disease through a test (i.e., LR+ > 10 = large; 5 > LR+ > 10 = moderate; 2 > LR+ > 5 = small; 1 > LR+ > 2 = minimal; 0 > LR+ > 1 = no increase). Available scientific evidence regarding each of the topics was graded in terms of level of confidence (LoC: VL = very low, L = low, M = moderate, and H = high), as reported by the GRADE collaboration [74, 75]. In the GRADE system, valid diagnostic accuracy studies can provide high quality evidence of test accuracy. Quality of evidence (GRADE) for each study was evaluated based on LR+ values, LR+ probability increase, and the size of the sample included for each study (i.e., e.g., a study with a moderate LR+ probability increase but with a relatively small sample (*n* = 20) would be rated as low in terms of quality of evidence). (See Tables 1, 2, and 3.)

**Figure 1:**
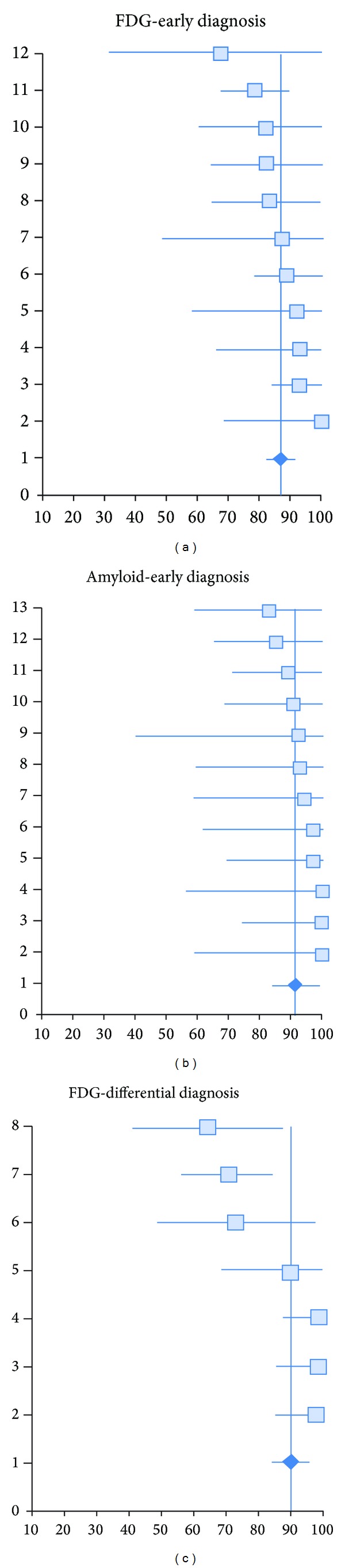
Forest plots of sensitivity measures and 95% confidence intervals for individual studies included in each meta-analysis.

In addition, we obtained a summary measure of effectiveness in each meta-analysis by weighting individual study effect measures according to their variance and by adopting a general inverse-variance weighted fixed-effects model to summarize individual effect measures (i.e., sensitivity analysis) and a *Q* test was performed to measure heterogeneity among studies. Sensitivity measures for each study were then arranged in a forest plot together with their 95% confidence intervals In order to represent the position of each study included over the central tendency, represented by the calculated summary fixed-effect sensitivity measure (see Figures 1(a), 1(b), and 1(c)).

### 2.3. Qualitative versus Quantitative Assessment

A description of differences in the levels of sensitivity and specificity for the standard visual FDG-PET scan or dichotomous readout based amyloid- PET with respect to parametric or semiquantitative analysis was performed on the basis of the data in literature reporting sensitivity and specificity of both the visual and the parametric methods in the same population.

## 3. Results

### 3.1. ^18^F-FDG PET in the Early Diagnosis of AD

The systematic review identified a total of 10 studies that met our inclusion criteria (see Table 1); the most relevant findings were as follows.

Arnáiz et al. [79] showed that, in a cohort of *N* = 20 MCI followed for a mean observational period of 36 months, reduced glucose metabolism from left temporoparietal area could predict conversion with a 75% percentage of correct classification, resulting in 67% sensitivity and 82% specificity. Authors conclude that these measures of temporoparietal metabolism may aid (together also with neuropsychological data) in predicting evolution of MCI patients to AD.

In a landmark study, Herholz and colleagues [42] investigated metabolic abnormalities with ^18^F-FDG-PET in a cohort of *N* = 110 HC and *N* = 395 probable AD. Despite the cross-sectional nature of the study, useful information was provided about an early diagnosis of AD because of the fragmentation of the pAD group in different subgroups related to probable disease severity (e.g., very mild probable AD group, MMSE ≥24). Authors calculated an AD t-sum score for each individual, and this score was applied to discriminate between various subgroups and controls. This method yielded 93% sensitivity and 93% specificity in classification of pAD versus HC, acting as a very useful tool to early diagnosis of AD.

Similarly, Mosconi and colleagues [80] followed a group of *N* = 37 MCI patients for a 12-month period. At the followup, *N* = 8 MCI converted while *N* = 29 remained stable. Authors analyzed, with a voxel-based method and analysis of variance, regional differences in cerebral glucose metabolism, using conversion (*y*/*n*) as outcome and APOE genotype (E4+/E4−) as grouping factor. Results show that for the whole MCI sample, inferior parietal cortex hypometabolism could predict conversion to AD with 84% diagnostic accuracy, 100% sensitivity, and 95% specificity. Furthermore, E4 carriers (E4+) converters (*N* = 5) presented significantly decreased metabolism in frontal areas, such as anterior cingulate cortex (ACC) and inferior frontal cortex (IFC). The authors' conclusion is that ^18^F-FDG-PET may improve prediction of the MCI-AD conversion especially when combined with APOE genotype information.

Anchisi and coworkers [17] investigated in a longitudinal study a cohort of *N* = 67 amnestic-MCI patients of which *N* = 48 underwent follow-up examination at a (at least) 12-month interval. The ROC curve calculated for the glucose metabolism measured in two voxel ROIs (posterior cingulate and temporoparietal) showed an area under the curve (AUC) of 0.0863. With a cut-off at 1.138, authors reported 92.9% and 82.4% as, respectively, sensitivity and specificity in discriminating converters versus nonconverters. In addition, negative predictive value of 96.55% and a positive predictive value of 68.4% were reported. Furthermore, authors combined functional metabolism impairment with memory test score (Long free delay recall part of the California verbal learning test, CVLT-LFDR) [81] showing an inverse pattern: lower sensitivity (85.7%), higher specificity (97.1%), lower negative predictive value (94.3%), and a higher positive predictive value (92.3%). Authors claim that using ^18^F-FDG-PET may help in predicting short-term conversion to AD, particularly combined with memory scores and also to account for the functional heterogeneity among subjects with aMCI.

Drzezga and coworkers [82] in a longitudinal prospective study on 30 MCI patients (mean observation period, 16 months) assessed the value of FDG-PET in detecting brain metabolic abnormalities in early AD, by using Neurostat [83] to perform an observer-independent statistical comparison with an age-matched reference database. The authors reported that the sensitivity and specificity of FDG-PET with regard to early diagnosis of AD in MCI patients were 92% and 89%, respectively.

Haense et al. [84] also investigated performance of ^18^F-FDG-PET for detection of AD within two different samples, from ADNI and Network for Standardisation of Dementia Diagnosis (NEST-DD). The cohort from ADNI consisted in *N* = 102 HC and *N* = 89 AD, while the sample from NEST-DD comprised *N* = 36 HC and *N* = 237 AD. The authors generated AD t-sum maps and used a preset cut point for discrimination. Results were twofold: (1) AD presented much higher AD t-sum maps than HC in both samples and (2) early onset-AD presented higher AD t-sum maps than late-onset AD. The cut-off threshold yielded sensitivity and specificity of 83% and 78%, respectively, in ADNI; in NEST-DD, results showed 78% sensitivity and 94% specificity. Authors conclude that this automated procedure to analyze ^18^F-FDG-PET scans is useful for the discrimination and is also more accurate for early onset AD.

Yuan and colleagues [20] performed a meta-analysis to evaluate and compare the ability of FDG-PET, single-photon emission tomography (SPECT), and structural MR imaging to predict conversion to AD in patients with MCI. Relevant studies were identified with MEDLINE from January 1990 to April 2008 and a meta-regression was carried out from eligible studies on the diagnostic performance data for each technique. This study included data from 1112 MCI patients (of which *N* = 280 investigated by FDG-PET) and showed that FDG-PET had better concordance with follow-up results for the prediction of conversion to AD dementia. Approximately 88.9% of the patients with progressive MCI were detected as positive by FDG-PET, whereas 84.9% of stable patients had negative FDG-PET at first scanning time (sensitivity 88.9%, specificity 84.9%). Further, FDG-PET was found to perform better than SPECT and structural MR imaging in the prediction of conversion to AD in patients with MCI.

Recently, Landau and coworkers [85] compared different biomarkers of conversion and decline in MCI investigating a fairly large cohort throughout the different predictors (FDG-PET, MRI/hippocampal volume, CSF biomarkers, Memory Score/Rey Auditory Verbal Learning Test). As regards ^18^F-FDG-PET, *N* = 85 MCI were followed for a period of (mean) 21 months. During the observation period, *N* = 28 converted (MCIc) while *N* = 57 remained stable (MCIs). To evaluate the power of the prediction with ^18^F-FDG-PET measured metabolism (parametrically analyzed with SPM, metaROI global index), the authors obtained cut-off scores from an independent sample rather than using cut-off scores present in literature. To do so, *N* = 102 Healthy controls and *N* = 97 AD were screened, resulting in a cut-off set at 1.21 to discriminate between “AD(+)” and “AD(−)”. ROC curve at this score showed 82% sensitivity, 70% specificity and an overall accuracy of 76% in discriminating between AD and controls. Thereafter, the derived cut-off was used to calculate predictive values of conversion for the MCI group, resulting in a positive predictive value of 41% and a negative predictive value of 78%. To say, the 78% of MCI classified as “AD(−)” at baseline remained stable, whereas MCI classified as “AD(+)” had a 2.72 greater risk of conversion. Then authors concluded that the FDG-PET was the most informative biomarker, especially when combined with RAVLT episodic memory score.

In a longitudinal study comparing ^11^C-PIB-PET, ^18^F-FDG-PET and MRI, Brück and coworkers [86] investigated MCI conversion in a sample of *N* = 29 MCI (of which, only *N* = 22 underwent also ^18^F-FDG). Clinical follow-up was carried on at a 24-months interval. During the observation period *N* = 13 MCI converted to AD while *N* = 9 MCI remained stable. All the ^18^F-FDG-PET were optimized and analyzed with region of interest approach and SPM methodology, deriving a cut-off of 1.16 in left lateral temporal cortex (internally derived). This cut point was used to classify patients in “High” and “Low” ^18^F-FDG, resulting in a sensitivity of 87% and a specificity of 78% in predicting conversion to AD. Similarly, patients were divided in “High” and “Low” ^11^C-PIB depending on PiB uptake in lateral frontal cortex (internally derived cut-off: 1.57), providing 65% sensitivity and 75% specificity. When combined, ^18^F-FDG and ^11^C-PIB (e.g., Low FDG-High PiB) resulted in 87.5% sensitivity and 71.4% specificity. The authors' claim is that ^18^F-FDG and ^11^C-PIB are better than hippocampal volume in predicting conversion.

Arbizu and colleagues very recently [87] proposed a new score for automated analysis of ^18^F-FDG-PET, called AD-Conv score, as a tool for single-subject prediction of conversion to AD. Their cohort comprised *N* = 80 HC, *N* = 121 MCI of which *N* = 36 MCIc (at 18-months interval) and *N* = 85 MCIs (at 24-months interval) and *N* = 67 AD. Briefly, their method consisted in generating an “AD-PET-pattern” from an external reference population and based on z-score map obtained with SPM. This map was then compared with individual hypometabolism voxel-by-voxel resulting in an AD-PET-index, that combined with age and gender generated the AD-Score. Starting from this score, meant to discriminate between AD and HC, authors generated a score to discriminate between MCIc and MCIs applying several modifications. First, instead of using a whole brain z-map (the AD-PET-pattern), AD-PET index was segmented in five volumes-of-interest (VOIs), namely left parietal, right parietal, left temporal, right temporal and posterior cingulate, and then compared with individual hypometabolism resulting in the MCI-PET-Index. Furthermore, to compute the score, APOE genotype (E4+/E4−), years of education and MMSE were combined with age obtaining the AD-Conv-Score. Further statistical analysis showed that only hypometabolism in posterior cingulate area was significant in differentiating MCIc from MCIs and, together with APOE4 genotype and MMSE, yielded the AD-Conv-Score parameter. With an AD-Conv cut-off score at 0.28, the method classified MCIc and MCIs with 91.7% sensitivity and 62.4% specificity. As regards predictive values, a positive predictive value of 51% and a negative predictive value of 95% were shown.

### 3.2. ^18^F-FDG PET in Differential Diagnosis between Forms of Dementia

A total of 4 papers addressing the discrimination power of FDG-PET between different neurodegenerative forms met the criteria outlined above (see Table 2). Among the studies pinpointed in Table 2, three studies included patients with a clinical diagnosis of probable AD, three studies included patients diagnosed with Lewy-Body Dementia (LBD), and two studies included patients with a diagnosis of Frontotemporal lobar degeneration (FTLD).

Minoshima et al. [42] examined brain glucose metabolism of DLB and AD and showed that FDG-PET discriminates DLB from AD with 90% sensitivity and 80% specificity using autopsy confirmation. They also concluded that the presence of occipital hypometabolism preceded some clinical features of DLB and that FDG-PET sensitivity was superior in differentiating DLB from AD with respect to medical charts exclusively based on clinical diagnostic criteria.

Similarly, Gilman and coworkers [88] investigated metabolism differences between AD and DLB measured with ^18^F-FDG-PET in a sample of *N* = 25 AD, *N* = 20 DLB and *N* = 19 elderly HC. ^18^F-FDG scans were analyzed with Minoshima method on selected VOIs (global cortex and occipital cortex, known to discriminate between DLB and AD in terms of CMRglc). Furthermore discrimination power was estimated also for neuropsychological scores such as MMSE, confrontation naming test and verbal fluencies. Logistic regression showed that glucose metabolism in BA17 (visual cortex) presented 64.3% sensitivity and 65.2% specificity for diagnosis of DLB. To say, the hypometabolism patterns of these two diseases were similar except for the metabolic rate in visual cortex.

In the widely cited study by Foster et al. [33] the utility of ^18^F-FDG statistical parametric maps rather than simple transaxial FDG-PET scans for dementia diagnosis was evaluated. Six experienced raters were forced to make a diagnosis about a cohort of *N* = 45 patients, all pathologically confirmed, of which *N* = 31 AD and *N* = 14 FTD. Results showed that the utilization of ^18^F-FDG statistical maps (stereotactic surface projection maps SSP) yielded high diagnostic accuracy (89.6%), showing 73% sensitivity and 97.6% specificity. Authors conclude that also after a brief training in visual interpretation of ^18^F-FDG statistical maps this method is more reliable and accurate than clinical methods alone.

Mosconi and colleagues [89], in a large multicenter study, examined FDG-PET measures in the differentiation of AD, FTD, and DLB from normal aging and from each other (*N* = 548 subjects, including 111 healthy individuals). Each PET scan was Z-transformed by using automated voxel-based comparison resulting in statistical maps of disease-specific patterns of brain ^18^F-FDG uptake. The differentiation and classification of patients in independent groups between patients and controls and among dementia forms yielded 99% sensitivity, 65% specificity (97% accuracy) for AD compared with FTD; 99% sensitivity, 71% specificity (97% accuracy) for AD compared to DLB; 99% sensitivity, 98% specificity (98% accuracy) for differentiating between AD and healthy controls; 71% sensitivity, 65% specificity (68% accuracy) for DLB with respect to FTD. Thus, this study strongly supported the validity and diagnostic accuracy of FDG-PET in the differential diagnosis of the three major neurodegenerative disorders.

### 3.3. FDG-PET Summary

These data provide strong evidence for FDG-PET parametric imaging to detect pathological changes occurring in the brain. FDG-PET holds great promise for diagnostic assessment of patients with Alzheimer disease (AD) and the other two major neurodegenerative diseases (i.e., DLB and FTLD) to the point that the recently revised diagnostic criteria of AD [5, 9] as well as the new National Institute of Aging-Alzheimer Association criteria of MCI due to AD [6] for the first time recognize the specific role of FDG-PET as a topographical functional biomarker in Alzheimer disease definition. What is especially relevant in this context is that FDG-PET as a neurodegeneration biomarker has been placed before brain atrophy in specific regions, as shown by means of MRI, in the hypothetical cascade model of AD biomarkers [46]. In fact, FDG-PET maps distribution of glucose metabolism occurring mainly at synaptic level [90]. Thus, pathologic phenomena leading to neuritic dysfunction affects synaptic glucose consumption prior of causing cell death and detectable atrophy [91, 92]. As such, FDG-PET is a proxy of reduced glucose utilization at synaptic level of still alive neurons.

It must be acknowledged that voxel-based procedures for objective image analysis can now be easily applied clearly providing evidence for a role of FDG-PET in assessment of dementia through the identification of disease-specific hypometabolic patterns. The main advantages of automatic methods consist in the fact that images can be interpreted even by intermediate-skilled readers and that false positive results are virtually eliminated, thus increasing specificity.

The primary objective of both tabulated surveys was to select studies on the basis of the mandatory need for the evaluation of the FDG-PET scans based on an automatic, unbiased voxel-based analysis in order to achieve higher confidence in diagnostic accuracy to significantly reduce the gap with post-mortem gold standard confirmatory diagnosis. The evidence provided in the tabulated surveys supports the role of FDG-PET as an effective tool aiding in the early diagnosis and differential diagnosis of dementia. The diagnostic accuracy of FDG-PET resulted to be high also in subjects with prodromal disease, for whom the clinical diagnosis and differential diagnosis are especially challenging. In fact, [1] claimed that “the sensitivity and specificity available with FDG-PET near the time of initial diagnosis of AD is similar to longitudinal clinical diagnosis over 3-4 years”.

### 3.4. Amyloid-PET in the Diagnosis of AD

The systematic review identified a total of 12 studies that met our inclusion criteria (see Table 3); the most relevant findings were as follows.

In their study, Rowe and coworkers [93], investigated the reliability of the ^18^F-BAY94-9172 (Florbetaben) in a relatively small cohort (*N* = 15 AD, *N* = 15 HC and *N* = 5 FTD) in discriminating between the three conditions. Authors analyzed quantitatively the neocortex uptake with SUVR measure, using the cerebellum as reference region. Experienced raters then visually inspected the maps of SUVR distributions. Visual inspection of SUVR maps yielded 100% sensitivity and 90% specificity in discriminating AD versus HC or FTLD. Authors conclude that florbetaben imaging can be included successfully in clinical use.

Using ^18^F-Flutemetamol PET scan in 25 HC, 20 MCI and 37 AD patients, Vandenberghe et al. [94] using SUVR distributions showed 93.1% sensitivity and 93.3% specificity and a very high correlation with ^11^C-PIB uptake (*r* = .89) for visual inspection. It is noteworthy that sensitivities and specificities did not differ significantly between qualitative (visual) and quantitative methods (SUVR cutoff automated classification in raised uptake category). Further, it has been shown that the tracer uptake highly correlated with percentage of brain area of amyloid measured by cortical biopsy [95].

Barthel and colleagues [96, 97] investigated the use of ^18^F-Florbetaben (^18^F-BAY94-9172) PET analysis in two contiguous studies (phase 0 and 2) involving 69 HC and 81 AD patients and found that visual assessment of PET images allowed 80% sensitivity and 91% specificity. On the other side, linear discriminant analysis of regional SUVR yielded an 85% sensitivity and 91% specificity. The same tracer has been demonstrated to be useful in discriminating different forms of dementia as well as patients from controls [12, 93]. The first results on florbetaben indicate that this radiopharmaceutical, while having a narrower dynamic range than ^11^C-PiB PET, is able to clearly differentiate HC from AD patients with a comparable effect size [98]. Moreover, quantification of *β*-amyloid binding from florbetaben PET data is feasible and all *β*-amyloid binding parameters including SUVR are excellent in discriminating between *β*-amyloid positive and negative scans [99].

In the study by Rostomian et al. [58], ^18^F-FDG and ^11^C-PIB were compared to evaluate the power of diagnosis of the* in vivo* imaging of fibrillar beta-amyloid versus metabolism or CSF. The authors tried first in a test cohort composed by *N* = 10 patients with various clinical diagnosis and, when identified the correct iterative algorithm, analyzed a sample of *N* = 42 AD and *N* = 31 FTLD with both FDG-PET and C-PIB PET (these map were obtained from *t*-test with reference regions, such as cerebellar for PiB). Results showed that with PIB PET had 90.5% sensitivity and 83.9% specificity (for AD), versus the, respectively, 88.1% and 83.9% with FDG-PET. Temporal pole and neocortex was significant for both the compounds, whereas the frontal lobe was particularly significant for PIB-PET. Authors conclude that the combined use of these two compounds can be very useful for early diagnosis of AD.

Other amyloid-PET studies addressing AD and MCI cases in large series came out in the literature reporting high sensitivity and intermediate/low values of specificity [21, 46, 62, 100, 101].

In the study by Villemagne et al. [12] authors still evaluated ^18^F-Florbetaben in imaging AD versus other dementia types. Their cohort consisted in *N* = 32 HC, *N* = 20 MCI, *N* = 30 AD, *N* = 11 FTD, *N* = 5 LBD, *N* = 5 Parkinson's Disease (PD) and *N* = 4 Vascular Dementia (VaD). SUVR values for whole brain neocortical retention were calculated using cerebellar cortex as reference region. Results showed that almost all of the AD group (96%) and more than half of the MCI group (60%) presented diffuse cortical retention whereas the other groups presented far minor cortical retention (FTLD = 9%, VaD = 25%, DLB = 29%, PD = 0%, HC = 16%). Semiquantitative SUVR analysis yielded a 97% sensitivity and 84% specificity in discriminating AD versus Healthy Controls. Authors conclude that ^18^F-Florbetaben can be useful in distinguishing AD from other dementias (e.g., FTLD) and that its effectiveness is comparable with the results obtained by ^11^C-PiB compound.

In a prospective cohort study by Clark et al. aimed to compare florbetapir PET with neuropathology at autopsy for detecting neuritic amiloid-*β* plaques, also the relation between SUVR and neuritic plaque density was assessed [102]. Based on values from a series of young participants who were cognitively normal, Joshi et al. [73] had previously proposed a cutoff value of 1,10 to distinguish normal from abnormal scans. In the paper of Clark et al., all the cases with no or sparse plaques at autopsy had SUVR values of less than 1,10, and all but one with moderate or frequent plaques at autopsy had SUVR values greater than 1,10. SUVR analysis showed a 97% sensitivity and 99% specificity in detecting high or low burden of amyloid plaques with a 24-months autopsy reference.

Using PET with florbetapir to quantify brain amyloid load in a routine clinical environment to differentiate between patients with mild to moderate AD and MCI from HC, the quantitative assessment of the global cortex SUVR reached a sensitivity of 92.3% and specificity of 90.5% with a cut-off value of 1.12 [29].

### 3.5. Amyloid-PET Summary

Up to date, the literature demonstrates that ^11^C-PiB PET allows reliable detection and in particular quantification of *β*-amyloid deposition in patients with AD.

However, because of the short half-life of ^11^Carbon, which requires an on-site cyclotron and radiochemistry laboratory, ^11^C-PiB has been compared with ^18^F-labeled tracers like ^18^F-Florbetapir, ^18^F-Flutemetamol or ^18^F-Florbetaben, which can be produced at central cyclotron and then delivered to clinical PET centers.

^18^F-Florbetapir and ^18^F-Flutemetamol are FDA approved in the US for clinical use, now also ^18^F-Florbetapir by the EMA, whereas ^18^F-florbetaben has not yet been approved in USA and Europe. These tracers could be largely used in detecting *β*-amyloid deposition and in distinguishing patients with AD from Frontotemporal dementia. As a limit, lipophilic plasma metabolites, which have been partially reported for ^18^F-labeled tracers, could increase non-specific background activity.

The results of these included studies show a promising role of those ^18^F-labeled tracers, but further data on larger number of patients also evaluated longitudinally are needed to clarify their diagnostic and prognostic potential roles in AD.

A central issue in PET estimation of amyloid load regards the use of semiquantitative analyses of images. In this view, a consensus regarding categorization of positive and negative subjects has not been established so far. For example, some groups have treated SUVR as a continuous variable whereas other groups have dichotomized subjects into positive and negative groups using a cut-off score, since the distribution of this variable is usually skewed. Further, there is variability in categorization approaches amongst studies that dichotomize into positive and negative groups. Some authors considered positive those subjects showing SUVR values that are 1, 1.5 or 2 standard deviations higher than normal controls [34, 56, 103–105], while others used more complex approaches such as cluster analyses [12, 48, 106, 107], iterative outlier removal [108] or complex functions [94]. SUVR cut-off values separating negative from positive subjects vary in the literature from 1.1 to 1.6, with a mean value around 1.3. The limit of classifying into positive and negative subjects relies on the fact that the threshold is often dependent on the distribution of SUVR values present in the control group under investigation rather than on a group of subjects lacking A*β* deposition.

In a recent study, ^11^C-PiB and florbetapir PET were compared in a retrospective sample of cognitively normal older controls, patients with MCI, and patients with AD. ^11^C-PiB and florbetapir retention ratios were strongly associated in the same individuals, and the relationship was consistent across several data analysis methods, despite scan-rescan intervals of more than a year. The findings of this study indicate that cutoff thresholds for determining positive or negative amyloid-*β* status can be reliably transformed from PIB to florbetapir units or vice versa using a population scanned with both radiopharmaceuticals [71].

Nordberg et al. [22] in a European multicentre PET study of fibrillar amyloid in AD based on very large datasets demonstrated the robustness of [^11^C]-PIB PET as a marker of neocortical fibrillar amyloid deposition in brain when assessed in a multicentre setting. The variance of [^11^C]PIB retention between different participating centers was low compared to the large differences between diagnostic groups, suggesting that results obtained from [^11^C]PIB PET are highly consistent and reproducible. MCI PIB-positive patients showed more severe memory impairment than MCI PIB-negative patients and progressed to AD at an estimated rate of 25% per year. None of the MCI PIB-negative patients converted to AD, and thus PIB negativity had a 100% negative predictive value for progression to AD. This supports the notion that PIB-positive scans in MCI patients are an indicator of prodromal AD and that amyloid imaging is both a highly useful tool for diagnosis of AD in its earliest symptomatic stages and is suitable for identifying patients for antiamyloid therapy in multicentre clinical trials. The paper reports also the vast majority of healthy controls (46 out of 51) and showed neocortical [^11^C]PIB retention ratios in the very narrow range of 1.13 to 1.39 (mean 1.26 ± 0.07). The upper 95% confidence limit in the normally distributed control population was 1.41, thus defining the normal limit.

One of the main issues since the advent of amyloid tracers remains and is represented by a percentage of HC showing an amyloid load in the range of patients with AD [22, 107, 109]. One of the future challenges in PET studies with ^18^F amyloid tracers is to reach standardize quantitative measures (especially by means of longitudinal approaches) in order to establish reliable quantitative cut-offs that can be helpful in separating HC and AD subjects, in differential diagnosis of dementia and in providing prognostic indices for those subjects showing early signs of cognitive loss.

### 3.6. Qualitative versus Quantitative Assessment

Few papers in literature systematically investigated improvements in diagnostic accuracy and/or in differential diagnosis obtained by using quantified (or semiquantified) and qualitative analysis of FDG-PET scans. The results showed that the qualitative interpretation by visual reading of brain ^18^F-FDG-PET scans and amyloid-PET scans clearly lacks clear-cut milestones to distinguish between a normal and a pathological scan. Indeed, in the already cited study by Foster and coworkers [33], authors compared five separate methods (clinical summaries, diagnostic checklist alone, summary and checklist, transaxial ^18^F-FDG-PET scans and ^18^F-FDG-PET stereotactic surface projection metabolic and statistical maps-SSP) for distinguishing AD from FTD in an autopsy-referenced cohort of *N* = 31 AD and M = 14 FTD, adopted by six dementia experts. Data showed that the transaxial FDG-PET scans method yielded 96% sensitivity, 59% specificity and a mean accuracy of 84.8% in distinguishing AD versus FTD. On the other hand, the ^18^F-FDG-PET SSP method improved sensitivity (97.6%), specificity (73.2%) and overall accuracy (89.2%). Authors conclude that ^18^F-FDG-PET improves dementia diagnosis accuracy, especially when metabolism was quantitatively analyzed prior to visual expert rating and interpretation.

Recently, Rabinovici et al. [34] compared ^11^C-PiB and ^18^F-FDG in differential diagnosis of AD and FTLD in a cohort of *N* = 62 AD and *N* = 45 FTLD. It is noteworthy that the authors compared also qualitative (visual) and quantitative (DVR for ^11^C-PiB, cut-off at 1.2 and regional ROI Z-score for ^18^F-FDG) methods in their diagnostic efficacy. As regards qualitative evaluation of PET scans, ^11^C-PiB PET yielded higher sensitivity for AD (89.5% versus 77.5%) and slightly lower specificity (83% versus 84%). Quantitative thresholds for automated classification of scans provided interesting results. As a matter of fact, while ^11^C-PiB PET DVRs yielded very similar results (89% sensitivity 83% specificity versus 89.5% sensitivity and 83% specificity), quantitative analysis of ^18^F-FDG-PET increased specificity (98% versus. 84%). Authors conclude that with both methods ^11^C-PiB PET was more sensitive, while ^18^F-FDG-PET was more specific only when scans were interpreted quantitatively. Furthermore, a recent longitudinal study by Patterson et al. [35] showed that detection by Statistical Parametric Mapping (SPM) was more accurate (*N* = 18 subjects detected) than clinical evaluation of FDG-PET scans (*N* = 10 detected) in a cohort of *N* = 31 MCI followed for a 3-years period. Specifically, SPM detected correctly *N* = 9 MCI converters (versus *N* = 5 detected by subjective visual interpretation) and *N* = 4 subjects not meeting criteria for MCI (one of them was detected also visually), therefore highlighting a possible role for SPM in revealing metabolic defects anticipating clinical manifestations. Preliminary results in a study comparing inspection of visual FDG-uptake distribution maps and visual SPM hypometabolism maps in discrimination in a total cohort of *N* = 95 patients (*N* = 45 AD, *N* = 30 MCI, *N* = 25 FTLD) show higher sensitivity (96% versus 78%) and specificity (84% versus 50%) [110].

Other studies, even though not aiming as a primary endpoint to compare qualitative and quantitative analysis, provided results coherent with our claim. One of the most relevant findings is provided in the already cited study by Camus et al. [29] that investigated potential of ^18^F-Florbetapir in discriminating AD versus HC. Their results showed that while visual assessment yielded 84.6% sensitivity and a 38.1% specificity, a quantitative global cortex SUVR analysis yielded 92.3% sensitivity and 90.5% specificity, with a cutoff point set at 1.122.

### 3.7. Meta-Analysis and GRADE Analysis

Tables 1, 2, and 3 show the characteristics of each study included in each meta-analysis, namely population sample, method employed, follow-up in months (i.e., only for early diagnosis), sensitivity and specificity measures, LR+, LR+ probability of increase, and GRADE evaluation [76, 77]. The total number of patients summed across all studies for each meta-analysis was computed and included 1322 patients for FDG-early diagnosis, 647 for amyloid-early diagnosis, and 1011 for FDG-differential diagnosis. Summary sensitivity effect measures were .86 for FDG-early diagnosis, .91 for amyloid-early diagnosis, and .90 for FDG-differential diagnosis. *Q*-test values for FDG-early diagnosis (*Q* = 6,83) and for amyloid-early diagnosis (*Q* = 1,94) were below critical values assessed at *P* < 0.05, revealing low heterogeneity between studies included in each. The *Q*-value for studies included in the FDG-differential Diagnosis meta-analysis (*Q* = 18.61) was above critical values assessed at *P* < 0.05, indicating moderate heterogeneity. Forest plots for each meta-analysis show that the central tendency for the effectiveness of FDG-PET or amyloid-PET for the early or differential diagnosis of dementia is above 85%, however the 95% confidence intervals for studies FDG-early diagnosis reveal a lower degree of uncertainty with respect to amyloid-early diagnosis (see Figures 1(a) and 1(b)).

## 4. Discussion

Clinical, pathologic, and genetic evidence indicate that the primary dementias have different underlying aetiologies and pathogenetic mechanisms. Treatment approaches of these conditions are different and hopefully will be even more so in the future. Thus, accurate diagnosis is critical in order to maximize the efficacy and appropriateness of specific regimes. At present, best differential diagnosis of dementia relies on histopathological observations, usually available only at autopsy. When faced with a patient carrying a neurodegenerative disease possibly causing dementia, current guidelines suggest that the clinician must establish a probable etiopathogenic diagnosis based on evidence available from neurological and cognitive evaluation, blood tests, structural MRI neuroimaging, and PET imaging [5–8]. Attempts to differentiate between neurodegenerative diseases causing dementia based in the early prodromal phase can be hard, particularly when patients present with subtle prodromal symptoms or with clinical-neuropsychological characteristics that overlap between primary dementias or with an atypical profile of symptoms. Therefore, establishing valid and reliable markers of the main neurodegenerative diseases causing dementia which are capable to identify specific changes during the early clinical stages, or even in preclinical stages as it happens in genetic forms of AD, is a pivotal and strategical issue.

A decade ago, the American Academy of Neurology regarded CT and MR imaging as “optional” examinations for the diagnosis and evaluation of dementia [111]. This view was counterbalanced by a Consensus of the European Alzheimer Disease Consortium (EADC) in 2003, highlighting the changing philosophy on the role of neuroimaging in the dementia workup [112]. However, structural neuroimaging techniques, even if widely accepted and of high-value in the diagnosis and management, have no clear cut role in the very early stage of the diseases and at individual level. Attempts in measuring volumes of specific structures, such as the hippocampal formation, have been undertaken mainly in AD, with interesting results in group analysis, but still with lack of consistent and validated cut-off scores for individual analysis. In some neurodegenerative diseases other than AD, such as diffuse Lewy-body disease, MRI might present with multiple pattern of atrophy or even with null results in early stages. Thus, in the temporal dynamics of biomarkers in the Alzheimer's pathological cascade, atrophy represents the last phenomenon in comparison to biomarkers of brain dysfunction, early neurodegeneration, and amyloid deposition [46].

Functional neuroimaging techniques may aid in the early diagnosis of neurodegenerative disorders and to clearly support the final diagnosis. Positron emission topography (PET) allows the investigation of both the measurement of cerebral glucose metabolism by ^18^F-2-fluoro-2-deoxy-D-glucose (FDG) and the quantification of A*β* amyloid deposition through specific molecular imaging techniques involving radiopharmaceuticals binding to amyloid.

FDG-PET started to be used in AD about 30 years ago [37] but its role in the diagnostic road map of Alzheimer disease and related dementias has not gained general consensus up to few years ago. In fact, both the “Dubois” [5, 9] and the NIA-AA [6, 8] new diagnostic criteria have included FDG-PET as a valid tool for biomarker measure of neurodegeneration, by showing specific metabolic changes that precede atrophy as detected with MRI. The basic concept is that FDG-PET estimates glucose consumption at the synaptic-astrocyte level [90] thus picking-up very early changes already detectable even in asymptomatic subjects at high risk for AD [113, 114]. In AD, the core of such changes is the precuneus and the posterior cingulate cortex [17, 19], the MTL structures that are mainly highlighted with ROI-based than with voxel-based automatic approach, and the association posterior lateral parietal and temporal cortex. The same glucose utilization defect can be detected in other regions in FTLD [115, 116]; primary progressive aphasia (PPA) [117]; dementia with Lewy bodies (DLB) [88]. FDG-PET studies are therefore increasingly being used as an adjunct in the initial clinical evaluation of patients with suspected dementia, particularly to aid in early detection [17] or when clinical diagnosis is doubtful. As shown by the here included studies, voxel-based FDG-PET as* in vivo* biomarker measure plays a key role in the identification of early functional brain derangements. In this view, a recently introduced term designed to define the spectrum of cognitive function between healthy aging and dementia is mild cognitive impairment (MCI). It was [118] who first set out formal criteria for a diagnosis of MCI (subjective complaint of memory loss; objective impairment of ability; preserved general cognitive function; intact activities of daily living; individual does not meet criteria for dementia). People meeting these criteria are considered at higher risk of developing AD compared to general population [119]; consequently, MCI is considered the optimal clinical stage for both early detection and intervention of AD. More recently, the position paper by the International Working Group for New Research Criteria for the Diagnosis of AD [5] further introduced new concepts and distinguished between (i) preclinical states of AD, in which individuals are free of symptoms, yet have either biomarker evidence of Alzheimer's pathology or a monogenic form of AD and (ii) prodromal or predementia AD, referring to those clinically affected individuals who do not have dementia yet but are diagnosed to have AD on the basis of evidence of Alzheimer's pathology from biomarkers.

With regard to degenerative diseases such as AD, physicians' confidence in diagnosing dementia can be undermined by several factors such as young age of onset, high education level (where neuropsychological tests can fail to reveal a subtle, despite substantial, cognitive decline), atypical presentation, and presence of psychiatric or cognitive comorbidities. The information provided by FDG-PET can therefore satisfy a fundamental need not only as a disease confirmatory test (high sensitivity) but also as an exclusion test (high specificity), especially in the early stage of the disease.

On this regard, an international consortium of investigators argued that, due to its high sensitivity, a negative (i.e., normal) FDG-PET scan strongly favors a normal outcome at followup [1, 10].

Two decades of ^18^F-FDG-PET studies in neurodegenerative diseases provided evidence for specific metabolic patterns [3].

Teune and colleagues [2] in a large study focusing on patients who had an FDG-PET scan at an early disease stage (96 patients: 20 patients with Parkinson's disease (PD), 21 with multiple system atrophy (MSA), 17 with progressive supranuclear palsy (PSP), 10 with corticobasal degeneration (CBD), 6 with dementia with Lewy bodies (DLB), 15 with Alzheimer's disease (AD), and 7 with frontotemporal dementia (FTD)) summarized the typical metabolic dysfunction in the different diseases. Each patient received a retrospectively confirmed diagnosis according to strictly defined clinical research criteria. FDG-PET images of each patient group were analyzed and compared with healthy controls using statistical parametric mapping (SPM5). The authors concluded that a combined method, including clinical information and voxel-based analysis technique, can discriminate patient groups across a spectrum of various neurodegenerative brain diseases, also at early disease stages. This implies that an early and more accurate diagnosis in individual patients can be made by comparing each subject's statistical objective map of brain glucose metabolism with a validated disease-specific hypometabolic pattern arising in specific brain areas, naturally grounded in a detailed clinical frame.

In the context of initial diagnosis, the exclusionary role of FDG-PET is especially clear in younger subjects with a suspicion of neurodegenerative disease. The high specificity of FDG-PET in AD, FTLD, and DLB implies that a negative, or normal, scan in the presence of the suspicion of dementia makes a diagnosis of a neurodegenerative disease very unlikely.

Based on the specificity of functional imaging with ^18^F-FDG-PET that measures synaptic dysfunction in different networks, depending on the underlying pathology, and on the sufficiently large body of evidence in the literature, we strongly claim that ^18^F-FDG-PET should be considered an essential component of the diagnostic workup of early onset dementia.

With regard to amyloid-PET, its potential clinical usefulness is strictly based on the assumption that early cerebral amyloidosis is virtually always detected in subjects on the path of AD. Even if there are still controversies about the so-called “amyloid hypothesis” in the pathogenesis of AD [120], the fact remains that amyloidosis is practically a held prerequisite for the diagnosis of AD. Nowadays, probably no physician would be highly confident with the diagnosis of AD in a patient in whom cerebral amyloidosis has not been confirmed. According to the temporal biomarker cascade hypothesis [52], brain amyloidosis would be a very early phenomenon, already detectable many years before the onset of symptoms.

As for differential diagnosis, amyloid-PET is less useful for the identification of DLB because most patients with this disease show brain amyloidosis that cannot be distinguished from that of AD patients [120]. In clinical practice, when a subject is evaluated because of cognitive symptoms, even if subtle, the demonstration of high brain amyloid load should strongly suggest one of the two main forms of neurodegenerative disease with amyloidosis, that is, AD or DLB. The topographic pattern of amyloid deposition is similar in these two conditions, but the pattern of neurodegeneration harbors significant differences because glucose hypometabolism specifically and extensively affects the occipital lobes in DLB and just marginally in AD whereas MTL hypometabolism, which is the classical fingerprint of AD, is seldom found in DLB [121]. Still in doubtful cases, the demonstration of nigrostriatal dopamine transporter deficit leads to identifying DLB with high accuracy [122].

Further, at least in AD, brain amyloid deposition seems to be a very early phenomenon and rather rapidly reaches a “plateau” at the time cognitive deficits become detectable [123], thus mirroring A*β* 1–42 levels in cerebrospinal fluid [124]. As such, the amount of amyloid deposition, along with A*β* 1–42 levels in cerebrospinal fluid, should not be viewed as an accurate index of disease progression. As a matter of fact, there is evidence that cognitive decline is much more related to the markers of neurodegeneration rather than to severity of amyloidosis, thus arguing for a higher sensitivity of PET-FDG and CSF levels of Tau and Phospho-Tau.

In the literature, visual inspection of amyloid burden has been reported to parallel the accuracy by quantification of the uptake (e.g., SUVR; see [34]). Other results, however, reported different findings (see [29]). It is of note that this may be true when discriminating mild to moderate AD with conditions in which amyloid retentions are null or nonsignificant (e.g., FTLD spectrum). When comparing early stages of AD pathology (MCI versus AD or even preclinical AD conditions), the methods based on quantification or semiquantification acquire relevance and might become mandatory. Typically, when considering patterns of accumulations in MCI during a follow-up period, quantitative analysis shows their power to detect changes [125].

In addition, while the* in vivo* detection of A*β* amyloid is gaining ground in the diagnosis of AD especially in MCI patients, the meaning of a positive PET scan in nondemented patients remains yet unclear. In our opinion, quantitative amyloid-PET scans, better defining the amount of amyloid load in these individuals, can prevent a positive amyloid scan to become a* de facto* diagnosis of AD. A paper from Mintun and colleagues [126] focused on this aspect by using ^11^C-PiB PET scan in 41 nondemented subjects and 10 AD patients. Results showed that, globally, patients had greater uptake ratios, although 4 of the controls had cortical binding values that were comparable to those of AD patients, thus supporting the hypothesis that amyloid imaging could be used to detect preclinical stages of AD. A similar result has been described more recently by Mormino and coworkers [108] who found that the 15% of a large cohort of elderly HC showed positive ^11^C-PiB uptake ratios. The clinical significance of these observations is still unclear and only long-term follow-up studies can clarify it. On the basis of the data available to date, it appears that these apparently healthy subjects with high amyloid load are likely to be on the path of AD, although we still ignore the time span from amyloid deposition and onset of first cognitive symptoms [46]. There is strong debate about the fate of “healthy” controls who displayed a positive amyloid-PET scan as we still ignore the time needed for an asymptomatic subject with amyloidosis to develop cognitive signs/symptoms. The time span has been indicated in a modeling of AD in the order of 10 years [46], but how to predict this time on an a real individual basis is still unknown. Noteworthy, recent evidence in individuals at risk for developing AD showed significant amyloid burden in autosomal dominant familial AD, even 15-16 years prior expected/predicted symptoms onset [113, 127] or 17 years before in sporadic AD cases [128]. The “nun” study has demonstrated that at least some individuals die with high brain amyloid load, but without any cognitive symptom or sign [129]. The biological evidence of amyloid load in human brains extended to elderly health individuals. This also implies ethical issues regarding what to communicate to an healthy volunteer found to be amyloid positive during clinical trials [130].

But just in this context of brain amyloidosis without symptoms, the demonstration of early signs of neurodegeneration in specific sites using voxel-based FDG-PET would be of great value. Starting from the observation that FDG-PET can be positive several years before the onset of dementia [64, 65], it would be possible to narrow the time of uncertainty in asymptomatic subject with amyloidosis. In other words, cognitively normal subjects showing cerebral amyloidosis through PET amyloid tracers along with glucose hypometabolism at specific sites would be at very high risk of developing a dementia process within few years. On the other hand, in a symptomatic patient with a suspicion of early AD, it has been proposed that amyloid-PET should precede any other evaluation just after morphological MRI [131] as a positive scan would strongly support the diagnosis of AD, thus avoiding most of the other diagnostic procedures, while a negative amyloid-PET scan would lead to search for other causes. Of utmost importance is the possibility to scan with amyloid-PET subjects in the MCI stage which represents a significant step toward the selection of groups with earlier AD for clinical trials. This would avoid including patients with a misdiagnosis and give experimental drugs the chance to be tested at the very onset of symptoms instead of when the disease has been already too progressed. While the potential of amyloid-PET is not a matter of debate in research, its misuse in clinical sets needs a careful regulation in order to give a proper role and a specific clinical context to this technique. That is why, recently, the Society of Nuclear Medicine and Molecular Imaging and the Alzheimer's Association have jointly convened the Amyloid Imaging Task Force (AIT) and published the Appropriate Use Criteria for amyloid-PET [132, 133]. They provided the appropriate use criteria for Amy-PET in which the circumstances for executing Amy-PET are listed. According to those, Amy-PET will be appropriate for patients with persistent or progressive unexplained MCI, or satisfying core clinical criteria for possible AD (i.e., atypical clinical course or etiologically mixed presentation; for patients with atypically young-onset dementia). Crucially, the AIT also define the inappropriate use of amyloid-PET in the following conditions: (1) in patients with core clinical criteria for probable AD and with typical age of onset; (2) determination of dementia severity; (3) positive family history of dementia or presence of apolipoprotein E (APOE) *ε*4; (4) in patients with a subjective cognitive complaint that is unconfirmed on clinical evaluation; (5) as an alternative to genotyping for suspected autosomal mutation carriers; (6) in asymptomatic individuals; (7) nonmedical uses such as (legal, insurance coverage, or employment screening).

In conclusion, on the basis of the present survey and also on the meta-analyses and GRADE analysis, we showed that there is moderate quality evidence for the effects of both modalities of PET imaging (FDG and Amyloid) in the early diagnosis of AD and conversion prediction, and, equally, moderate quality evidence for the differential diagnosis of patients with AD and the other major neurodegenerative dementia (i.e., DLB and FTLD). The three meta-analyses conducted through the three categories of studies (early diagnosis, disease progression and differential diagnosis), as remarked in the Results section, yielded significant results. Summary sensitivity effect measures were 0.86 for ^18^F-FDG-PET (1322 cases), 0.91 for amyloid-PET (797 cases), and 0.90 for differential Diagnosis (1011 cases). Therefore, on the basis of the studies included in the present survey, amyloid-PET seems to be more sensitive than ^18^F-FDG-PET in early diagnosis of AD. It is of note that our analysis included a sample of patients investigated by ^18^F-FDG-PET larger than the cohort investigated by amyloid-PET. Hence, even if the effect measure is lower, we can interpret that result as more robust. In addition, the grade analysis classified more ^18^F-FDG-PET studies as M (moderate, *N* = 7) than for amyloid-PET (*N* = 5) that is coherent with the previous claim. Lastly, as anticipated in Results section, the 95% confidence intervals for ^18^F-FDG-PET early diagnosis and disease progression reveal a lower degree of uncertainty with respect to amyloid-PET early diagnosis (see Foster plots, Figure 1). For these reasons, we can definitely conclude that both the topographical and pathological PET markers are very accurate and sensitive to early diagnosis of AD, as well as to differential diagnosis with other dementia (e.g., FTD or DLB) when appropriate data analysis at single subject level is performed.

This survey and GRADE analysis show a good overall quality of evidence for PET functional (FDG) and molecular (amyloid) imaging in early and differential diagnosis of AD, on the basis of voxel-based or parametric data quantifications. This approach will allow net benefits in terms of diagnostic and prognostic value of the information provided by PET imaging considering its sensitivity and accuracy.

## Figures and Tables

**Table 1:** Summary of included 18F-FDG-PET for early diagnosis and conversion prediction, with LHR, increase in LHR+, GRADE, and population.

Authors	Population	Method	Cohort investigated	Follow-up (months)	Sensitivity	Specificity	LHR+	Increase in the LHR+	Quality of evidence (GRADE)
Arnáiz et al., 2001 [79]	20MCI	ROI	20	36	0.67	0.82	3.72	Small	L
*Herholz et al., 2002 [27]	110 HC; 395 pAD	AD t-sum	395	—	0.93	0.93	13.29	Large	M
Mosconi et al., 2004 [80]	37 MCI	SPM	37	12	1	0.9	10.00	Moderate	M
Drzezga et al., 2005 [82]	30 MCI	SPM + Minoshima	30	16	0.92	0.89	8.36	Moderate	M
Anchisi et al., 2005 [17]	48 aMCI	SPM	48	12	0.929	0.824	5.28	Moderate	M
*Haense et al., 2009a [84]	89 AD; 102 HC	AD t-sum	89	—	0.83	0.78	3.77	Small	L
*Haense et al., 2009b [84]	237 AD; 37 HC	AD t-sum	237	—	0.78	0.94	13.00	Large	M
Yuan et al., 2009 [20]	280 MCI	Meta-analysis	280	14.25	0.888	0.849	5.88	Moderate	M
*Landau et al., 2010 [85]	85 MCI; 97 AD; 102 HC	SPM + ROI	97	—	0.82	0.7	2.73	Small	L
Brück et al., 2013 [86]	22 MCI	SPM + ROI	22	24	0.87	0.78	3.95	Small	L
*Arbizu et al., 2013 [87]	80 HC; 36 MCIc; 85 MCIs; 67 AD	Automated voxel-based analytical method	67	—	0.818	0.86	5.84	Moderate	M

Total number of patients and healthy controls considered in the study. Method: quantitative method applied in the study. Cohort investigated: number of patients considered for sensitivity and specificity estimations. Followup: duration of observational period (for early diagnosis study). Sensitivity and specificity: results of the study. LHR+: likelihood ratio. Increase in the LHR+: increase in the probability of the likelihood of the disease. GRADE: results of GRADE evaluation. Quality of evidence was evaluated based on LHR+ values, LHR+ increase probability, and size of the sample included.

Abbreviations: pAD: probable Alzheimer's disease; MCI: mild cognitive impairment; aMCI: amnestic mild cognitive impairment; MCIc: MCI converters; MCIs: MCI stable; HC: healthy controls.

*Studies including early diagnosis of AD.

**Table 2:** Summary of the included PET studies for differential diagnosis, with LHR+, increase of the LHR+, and GRADE.

Authors	Population	Method	Cohort investigated	Sensitivity	Specificity	LHR+	Increase in the LHR+	Quality of evidence (GRADE)
Minoshima et al., 2001 [42]	AD + LBD	Minoshima	74	0.9	0.8	4.50	Small	L
Gilman et al., 2005 [88]	AD + LBD	VOI lCMRglc	45	0.643	0.652	1.85	Minimal	VL
Foster et al., 2007 [33]	AD + FTD	Minoshima	45	0.732	0.976	30.50	Large	M
Mosconi et al., 2008a [89]	AD + FTD	Minoshima	297	0.99	0.65	2.83	Small	L
Mosconi et al., 2008b [89]	AD + LBD	Minoshima	226	0.99	0.71	3.41	Small	L
Mosconi et al., 2008c [89]	AD + HC	Minoshima	199	0.99	0.98	49.50	Large	M
Mosconi et al., 2008d [89]	FTD + LBD	Minoshima	125	0.71	0.65	2.03	Small	L

Population: different dementias considered in the diagnosis. Method: quantitative method applied in the study. Cohort investigated: number of patients considered for sensitivity and specificity estimations in the discrimination. Sensitivity and specificity: results of the study data show potential of discrimination. LHR+: likelihood ratio. Increase in the LHR+: increase in the probability of the likelihood of the disease. GRADE: results of GRADE evaluation. Quality of evidence was evaluated based on LHR+ values, LHR+ increase probability, and size of the sample included.

Abbreviations: AD: Alzheimer's disease; LBD: Lewy body dementia; FTD: frontotemporal dementia; HC: healthy controls.

**Table 3:** Summary of the included amyloid-PET studies included with LHR and GRADE analysis.

Authors	Population	Method	Cohort investigated	Follow-up months	Sens.	Spec.	LHR+	Increase in the LHR+	Quality of evidence (GRADE)
Barthel et al., 2011 [96, 97]	81 AD; 69 HC	ROI SUVR analysis	81	—	0.85	0.91	9.44	Moderate	M
Rabinovici et al., 2011 [34]	62 AD; 45 FTD	ROI DVR analysis	107	12	0.89	0.83	5.24	Moderate	M
Rostomian et al., 2011 [58]	42 AD; 31 FTD	ROI DVR analysis	73	16	0.905	0.84	5.66	Moderate	M
Rowe et al., 2008 [93]	15 AD; 5 FTD; 15 HC	SUVR analysis	20	12	1	0.9	10.00	Moderate	L
Villemagne et al., 2011 [12]	30 AD; 20 MCI; 32 HC; 11 FTD; 7 LBD; 5 PD; 4 VaD	SUVR analysis	30	—	0.97	0.84	6.06	Moderate	M
Clark et al., 2012 [102]	5 MCI; 29 AD; 12 HC; 13 ODD	SUVR analysis	47	24	0.97	0.99	97.00	Large	M
Camus et al., 2012 [29]	13 AD; 12 MCI; 21 HC	SUVR + Visual	13	—	0.923	0.905	9.72	Moderate	VL
Koivunen et al., 2011 [62]	29 MCI; 13 HC	PiB retention analysis	29	24	0.94	0.42	1.62	Minimal	VL
Mosconi et al., 2009 [19]	31 MCI	ROI ratio SPM	31	32.16	0.93	0.76	3.88	Small	L
Forsberg et al., 2010 [100]	37 mild AD; 21 MCI	ROI ratio SPM	58	33	1	0.71	3.45	Small	L
Jack et al., 2010 [46]	53 MCI	DVR	53	20.4	0.83	0.46	1.54	Minimal	VL
Wolk et al., 2009 [101]	23 MCI	DVR SPM	23	21	1	0.56	2.27	Small	L

Population: total number of patients and healthy controls considered in the study. Method: quantitative method applied in the study. Cohort investigated: number of patients considered for sensitivity and specificity estimations. Followup: duration of observational period (for early diagnosis study). Sensitivity and specificity: results of the study. LHR+: likelihood ratio. Increase in the LHR+: increase in the probability of the likelihood of the disease. GRADE: results of GRADE evaluation. Quality of evidence was evaluated based on LHR+ values, LHR+ increase probability, and size of the sample included.

Abbreviations: AD: Alzheimer's disease; FTD: frontotemporal dementia; MCI: mild cognitive impairment; ODD: other dementia; LBD: Lewy body dementia; VaD: vascular dementia; HC: healthy controls.
